# Inflammation, Cancer and Oxidative Lipoxygenase Activity are Intimately Linked

**DOI:** 10.3390/cancers6031500

**Published:** 2014-07-17

**Authors:** Rosalina Wisastra, Frank J. Dekker

**Affiliations:** Pharmaceutical Gene Modulation, Groningen Research Institute of Pharmacy, University of Groningen, Antonius Deusinglaan 1, 9713 AV Groningen, The Netherlands; E-Mail: r.wisastra@gmail.com

**Keywords:** inflammation, cancer, oxidative stress, lipoxygenases, nuclear factor κB

## Abstract

Cancer and inflammation are intimately linked due to specific oxidative processes in the tumor microenvironment. Lipoxygenases are a versatile class of oxidative enzymes involved in arachidonic acid metabolism. An increasing number of arachidonic acid metabolites is being discovered and apart from their classically recognized pro-inflammatory effects, anti-inflammatory effects are also being described in recent years. Interestingly, these lipid mediators are involved in activation of pro-inflammatory signal transduction pathways such as the nuclear factor κB (NF-κB) pathway, which illustrates the intimate link between lipid signaling and transcription factor activation. The identification of the role of arachidonic acid metabolites in several inflammatory diseases led to a significant drug discovery effort around arachidonic acid metabolizing enzymes. However, to date success in this area has been limited. This might be attributed to the lack of selectivity of the developed inhibitors and to a lack of detailed understanding of the functional roles of arachidonic acid metabolites in inflammatory responses and cancer. This calls for a more detailed investigation of the activity of arachidonic acid metabolizing enzymes and development of more selective inhibitors.

## 1. Introduction

Inflammation and cancer are closely linked by specific oxidative processes in the tumor microenvironment [1]. Therefore, oxidative enzymes that are known to play a key role in inflammation are increasingly investigated in connection to cancer. The immune response on the cellular levels is carefully orchestrated by signal transduction pathways such as the nuclear factor κB (NF-κB) pathway. In this review we will discuss the lipid mediators that are produced by lipoxygenases, their role in the regulation of inflammatory responses among others via the NF-κB pathway, their connection with inflammatory diseases and cancer as well as small molecule lipoxygenase inhibitors.

## 2. Lipid Mediators Produced by Lipoxygenases

**Scheme 1:**
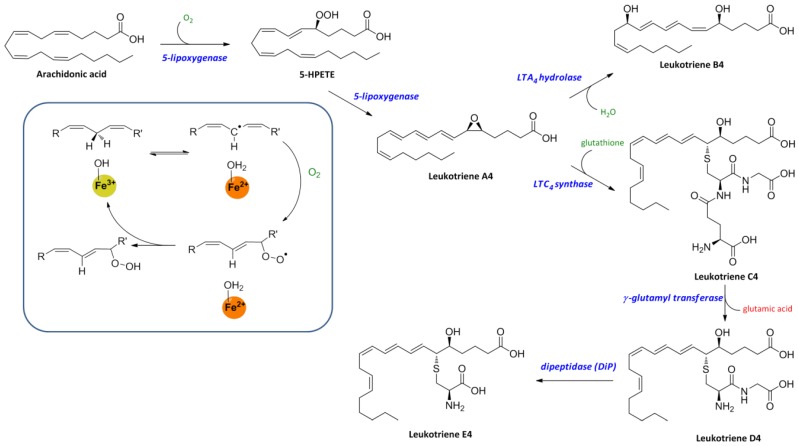
Oxidation reactions of lipoxygenases in the leukotriene (LT) biosynthesis pathways.

Lipoxygenases are a group of oxidative enzymes with a non-heme iron atom in their active site, which are involved in the regulation of inflammatory responses by generation of pro-inflammatory mediators known as leukotrienes or anti-inflammatory mediators known as lipoxins. These enzymes catalyze the insertion of oxygen (O_2_) into poly-unsaturated fatty acids (PUFAs) such as arachidonic acid and linoleic acid. It has been described that the catalytic reaction of lipoxygenases involves a single electron oxidation by the active site iron atom which switches between Fe^2+^ and Fe^3+^ redox states [2]. In the catalytic reaction, Fe^3+^ is reduced to Fe^2+^ with concomitant oxidation of the lipid substrate by hydrogen abstraction from a bis-allylic methylene to give a pentadienyl radical, which is re-arranged to provide a 1-cis,3-trans-conjugated diene moiety. Subsequently, a stereo-specific insertion of oxygen at the pentadienyl radical takes place to form an oxygen centered fatty acid hydroperoxide radical. The intermediate hydroperoxide radical is reduced to the corresponding anion with concomitant re-oxidation of iron to Fe^3+^ (Scheme 1) [3].

Lipoxygenases catalyze the formation of hydroperoxy eicosatetraenoic acids (HPETEs) from arachidonic acid. These HPETEs are subsequently reduced and transformed to form so called eicosanoids, which are signaling molecules that play an important regulatory role in the immune responses and other physiological processes. In general, lipoxygenases are classified as 5-, 8-, 12, and 15-lipoxygenases according to their selectivity to oxygenate fatty acids in a specific position [4]. The importance of fatty acids oxygenation by lipoxygenase enzymes has been described for many physiological processes (Table 1).

Lipoxygenases are commonly found in the plant and animal kingdoms. Although the overall architecture of plant lipoxygenases such as soybean lipoxygenase is similar to mammalian lipoxygenases, they share little sequence similarity (about 25%) [5]. In contrast, there are sequence similarities of about 60% among human 5-, 12- and 15-lipoxygenases [6]. Even though these enzymes show a high sequence similarity, the regulatory mechanism of 5-lipoxygenase (5-LOX) is more complex compared to the other human lipoxygenases. In general, lipoxygenases are comprised of two domains; N-terminal and C-terminal domains. The N-terminal domain is a regulatory domain and consists mostly of β-barrels, while the C-terminal domain is a catalytic domain and consists mostly of α-helices [6]. The non-heme iron atom is located in the catalytic C-terminal domain, whereas the function of the N-terminal domain is not unambiguously characterized. For 5-LOX, it is clear that the N-terminal domain is essential for translocation to the nuclear membrane whereas for the other LOXs, this is still under debate [6,7].

**Table 1:** Human lipoxygenases and their most important substrates, products, and functions.

Lipoxygenase	Substrate	Product	Physiologial function	Ref.
5-lipoxygenase (5-LOX)	arachidonic acid	5(S)-HPETE, Leukotriene A4	Pro-inflammatory mediator	[8]
	γ-linoleic acid	Dihomo-γ-linoleic acid (DGLA)	Inhibition of arachidonic acid conversion	[9]
	Eicosapentaenoic acid (EPA)	Leukotriene A5	Anti-inflammatory mediator/inhibitor LTA4 hydrolase	[10]
Platelet 12-lipoxygenase (p12-LOX)	arachidonic acid	12(S)-HPETE	Modulation of platelet aggregation	[11,12,13]
	Dihomo-γ-linoleic acid (DGLA)	12(S)-HPETrE		
	Eicosapentaenoic acid (EPA)	12(S)-HPEPE		
	α-linoleic acid	12(S)-HPOTrE		
12R-lipoxygenase (12R-LOX)	arachidonic acid	12(R)-HPETE	Epidermal barrier acquisition	[14]
	Linoleyl-ω-hydroxy ceramide	9(R)-hydroperoxyllinoleoyl-ω-hydroxy ceramide		
epidermis LOX3 (eLOX3)	9(R)-hydroperoxyllinoleoyl-ω-hydroxy ceramide	9(R)-10(R)-trans-epoxy-11E-13(R)-hydroxylinoleoyl-ω-hydroxy ceramide		
15-lipoxygenase-1 (15-LOX1)	linoleic acid	13(S)-HPODE	modulation of MAP kinase signaling pathways	[15,16,17]
	arachidonic acid	15(S)-HPETE	modulation of leukotriene B4, pro-inflammatory mediators	
15-lipoxygenase-2 (15-LOX2)	arachidonic acid	15(S)-HPETE	negative cell cycle regulator and tumor supressor	[18,19]

Human 5-LOX activity is influenced by the presence of Ca^2+^, which reversibly binds to the enzyme with maximum binding of two Ca^2+^ ions per 5-LOX. Ca^2+^ binding causes an increase in hydrophobicity, which promotes membrane association of 5-LOX [20]. Furthermore, the presence of adenosine tri-phosphate (ATP) appears to be important for optimal 5-LOX activity. It has been reported that 5-LOX has an ATP binding site, in which both the adenine-base and the phosphate moieties of ATP are essential for the activation. However, the stoichiometry, the affinity and the location of ATP binding on 5-LOX remain elusive [21]. In addition, the cellular 5-LOX activity is essentially dependent on a small membrane protein called five-lipoxygenase-activating protein (FLAP). Although, FLAP shares about 50% sequence similarity with human LTC4 synthase, its activity is not glutathione dependent. The influence of FLAP on 5-LOX activity is exerted via an allosteric mechanism. FLAP plays a role in arachidonic acid recruitment to 5-LOX and its conversion to 5-HPETE and LTA4 [22].

Two different types of 12-LOX have been identified based on the differences in tissue distribution, which are respectively named as platelet 12-LOX (p12-LOX) and 12R-LOX. Platelet 12-LOX is mostly found in platelets as a platelet aggregation modulator, whereas 12R-LOX is mostly found in skin cells in which it plays a role in epidermal barrier properties [13,14]. There are also two subtypes of 15-LOX, named as 15-LOX-1 and 15-LOX-2. 15-LOX-1 is highly expressed in leukocytes and airway endothelial cells [23,24] while, in contrast, 15-LOX-2 is expressed in prostate, lung, cornea, and many tissues such as liver, colon, kidney, spleen, ovary, and brain, but not in leukocytes [25] Moreover, cells induced by interleukin (IL)-4 and IL-13 show selective increase of 15-LOX-1 expression and not 15-LOX-2 [26]. A lack of similarity between 15-LOX-1 and 15-LOX-2 at the primary sequence level contributes to their distinct biological roles [27]. 

## 3. Biosynthesis of Leukotrienes: Initiation of Inflammatory Responses

Leukotrienes (LTs) received their name because they were found in various types of leukocytes, such as granulocytes, monocytes and mast cells. The pro-inflammatory leukotrienes are divided into two classes, dihydroxy acid leukotriene LTB4 and the cysteinyl leukotrienes LTC4, LTD4, and LTE4 [28].

Biosynthesis of leukotrienes is regulated by the activity of 5-lipoxygenase. Upon inflammatory stimulation, cytosolic phospholipase A2-α (cPLA2α) releases arachidonic acid from membrane lipids to start the leukotrienes biosynthesis. 5-lipoxygenase catalyzes the oxidation of arachidonic acid to 5-HPETE, which is subsequently converted into Leukotriene A4 (LTA4). LTA4, which is a LT precursor, is hydrolyzed by LTA4 hydrolase to form dihydroxy acid leukotriene LTB4. Another route is the conversion of LTA4 to cysteinyl leukotriene LTC4 by addition of a glutathione group by LTC4 synthase. Conversion of LTC4 by γ-glutamyl transferase results in LTD4 and glutamic acid release. Furthermore, dipetidase (DiP) breaks the amide bond in LTD4 to give LTE4 (Scheme 1).

**Figure 1:**
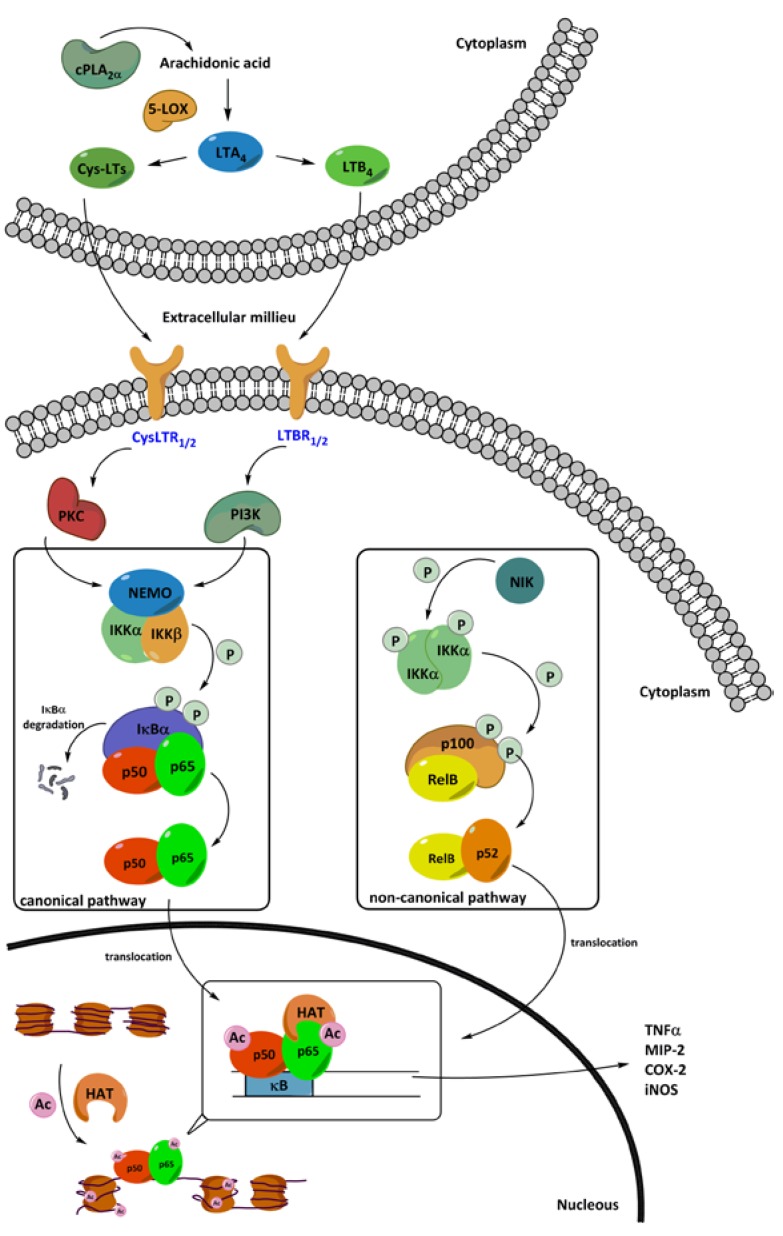
The roles of leukotrienes and acetylation in the expression of pro-inflammatory mediators through the NF-κB pathway. The activated cPLA2α produces arachidonic acid, which is further converted to LTA4 by the 5-LOX. LTA4 is then converted to LTB4 and cys-LTs and their binding to the leukotriene receptors activate the NF-κB pathway in leukocytes during inflammation. cPLA2α—cytosolic phospholipase A2-α; 5-LOX—5-lipoxygenase; LTA4—leukotriene A4; LTB4—leukotriene B4; Cys-LTs—cysteinyl leukotrienes; LTBR1/2—leukotriene B receptors 1 or 2; CysLTR1/2—cysteinyl leukotriene receptors 1 or 2; PI3K—phosphoinositide 3-kinase; PKC—protein kinase C; NEMO—NF-κB essential modulator; IκBα—inhibitor NF-κB; IKK—IκB kinase; NIK—NF-κB activation of inducing kinase; HAT—histone acetyltransferase. TNFα—tumor necrosis factor α; MIP—2-macrophage inflammatory protein-2; COX-2—cycloxygenas-2; iNOS—inducible nitric oxide synthase.

LTB4 has an important function as chemo-attractant and is also involved in the formation of reactive oxygen species. Binding of LTB4 to the Leukotriene B4 receptor 1 or 2 (LTBR1/2) activates the phosphatidylinostisol 3-kinase (PI3K) pathways [29]. In this way LTB4 is involved in the NF-κB pathway by stimulating the phosphorylation of IκBα, which results in activation of the NF-κB pathway. The cysteinyl leukotrienes LTC4, LTD4, and LTE4 activate two cysteinyl leukotriene receptors (CysLTR) 1 and 2, which also play a role in the regulation of NF-κB pathway [30] LTC4 induces the phosphorylation of NF-κB p65 and activates the NF-κB complex p50-p65. It also has been proposed that the LTC4 binding to the CycLT2 receptor will induce the phosphorylation of IκBα by involving protein kinase C (PKC) family enzymes (Figure 1) [31].

## 4. Nuclear Factor κB (NF-κB) in Inflammation

Among all the lipoxygenase products, leukotrienes have exceptional biological functions. A particular function is their action as pro-inflammatory mediators in the activation of the NF-κB pathway [32]. The nuclear factor κB (NF-κB) is an inducible transcription factor comprised of homo- and hetero-dimers of the NF-κB and Rel protein family [33] The NF-κB sub-family is comprised of two precursor proteins, p105 and p100, while the Rel sub-family is comprised of RelA/p65, RelB and c-Rel. p105 and p100 respectively are precursors of p50 and p52, which are transcription factors in the NF-κB pathways. The transcription factors of NF-κB are normally present in the cytoplasm in their inactive state in a complex with the inhibitory proteins of IκB family [33]. The production of pro- and anti-inflammatory mediators is highly correlated with gene expression through the NF-κB pathway [34]. There are two major pathways for NF-κB activation, the canonical pathway and the non-canonical pathway. In addition, an atypical pathway has also been identified. The heterodimer of RelA/p65and p50 is involved in the canonical pathway, whereas the heterodimer of RelB and p52 is involved in the non-canonical pathway [35,36]. The activated NF-κB pathway is involved in the pathogenesis of inflammatory diseases such as asthma, arthritis, inflammatory bowel diseases (IBD) and chronic obstructive pulmonary diseases (COPD) [37,38,39]. During inflammatory responses, both pro- and anti-inflammatory mediators are produced. The regulation of inflammatory responses relies on the careful orchestration of the expression of mediators that activate or suppress the immune response. 

### 4.1. The Canonical NF-κB Activation Pathway

Under normal conditions, the activity of the transcription factor complex RelA/p65-p50 is inhibited by its natural inhibitors, IκB proteins. Upon stimulation by pro-inflammatory cytokines such as TNFα and IL-1, IκB kinase (IKK) complex phosphorylates IκB proteins that cause the release of the RelA/p65-p50 dimer, which can subsequently translocate to the nucleus. The IKKs consist of the subunits IKKα, IKKβ and IKKγ, which is also known as the NF-κB essential modulator (NEMO) protein. In addition, the functions of RelA/p65 are also regulated by two group of enzymes; phosphoinositide 3-kinase (PI3K) and protein kinase B (PKB)/Akt kinases [40]. Kinases in the PI3K and PKB/Akt pathways induce the activation of IκB kinase to phosphorylate the IκB and stimulate the activation of transcription factors [41,42]. Furthermore, the phosphorylated IκBα protein is ubiquitinated and subsequently degraded [43]. Degradation of IκB leads to the translocation of the free p65-p50 dimer to the nucleus, in which p65-p50 then bind to the κB promoter regions and activates gene expression (Figure 1) [35,43]. 

### 4.2. The Non-Canonical NF-κB Activation Pathway

RelB in complex with p100 is present in the cytoplasm as inactive form of the transcription factor RelB-p52. The activation of the NF-κB via the non-canonical pathway is mediated by the IKK complex, which comprises two IKKα sub-units. The activation of the homodimer of IKKα is involving NF-κB activation of inducing kinase (NIK) and tumor necrosis factor receptor-associated factor (TRAF) [5,40]. Upon stimulation, the IKK complex is activated by NIK through a phosphorylation process, then the activated IKKα phosphorylates the inactive form of p100 subunit. Phosphorylation of p100 then leads to another post-translational modification; ubiquitination, which induces the proteolytic processing of p100 to form the active transcription factor p52. The formed heterodimer RelB-p52 is recruited to the nucleus to initiate the gene transcription (Figure 1) [44].

## 5. Role of Leukotrienes in Inflammatory Diseases

Over-expression of lipoxygenases and their pro-inflammatory products, leukotrienes, has been implicated in many human acute and chronic inflammatory diseases such as asthma, atherosclerosis, rheumatoid arthritis, inflammatory bowel diseases, dermatitis, and cancer. In some cases a connection between lipoxygenase activity and activation of the NF-κB pathway has been described (Table 2).

**Table 2:** Connection between lipoxygenase activity and NF-κB activity in specific diseases.

Disease	Observations	Ref.
Asthma	Ectopic expression of 15-LOX induces NF-κB mediated reporter gene expression in epithelial cells.	[45]
Cardiovascular diseases	Increased levels of 5-LOX metabolites in patients with atherosclerosis.	[46]
	The 15-LOX metabolite 15-HETE activates the NF-κB pathway and stimulates 15-LOX expression in a positive feedback loop.	[47]
Rheumatoid Arthritis	The 15-LOX metabolite 15-HETE increases IκBα degradation and activation of the NF-κB pathway.	[48]
Cancer	The 5-LOX metabolite LTB4 is capable of activating the transcription factor NF-κB in cancer cells	[49]

### 5.1. Asthma

Highly increased levels of LTC4, LTD4, and LTE4, which are 5-LOX metabolites, have been observed in lung tissues that were challenged with allergens. Up-regulation of these mediators is considered as the main cause of asthma since leukotrienes are potent regulators for smooth muscle contraction in bronchoconstriction. In addition, cysteinyl leukotrienes can cause plasma leakage from post-capillary venules in respiratory tissues, which can lead to inflammatory edema [50]. In addition, it has been shown that the expression of 15-LOX in lung epithelial cells activates the NF-κB pathway [45], which demonstrates a connection between LOX activity and NF-κB activation. These findings indicate that the modulation of the production of pro-inflammatory leukotrienes using small molecule inhibitors has potential for treatment of asthma.

### 5.2. Cardiovascular Diseases

Lipoxygenase activity has been implicated in the pathogenesis of cardiovascular diseases such as atherosclerosis. Lipoxygenases, as oxidative enzymes, are believed to have an important role in the oxidation of low density lipoproteins (LDLs) in macrophages to form foam cells [46]. The formed foam cells will develop plaques of atheroma and their accumulation in the arteries leads to atherosclerosis. In addition, an increase of the 5-LOX metabolites cysteinyl LTE4 levels in urine and LTB4 in the atheroma were observed in patients with atherosclerosis. In addition, it has been shown that the 15-LOX-1 and 15-LOX-2 metabolite 15-hydroxyeicosatetraenoic acid (15-HETE) promotes pulmonary artery inflammation via activation of the NF-κB pathway, which leads to increased expression of the 15-LOX enzymes in a positive feedback loop [47]. This demonstrates that inhibition of lipoxygenase activity can provide a treatment strategy for this cardiovascular disease.

### 5.3. Rheumatoid Arthritis

Since 5-lipoxygenase is the main catalyst for the formation of LTB4, its role in the development of rheumatoid arthritis becomes apparent with the identification of high LTB4 levels in the synovial fluid of arthritis patient [51]. This leukotriene is produced mainly by neutrophils, which are the most abundant leukocytes in rheumatoid joints [52]. A crucial role of LTB4 in arthritis induction and severity has been revealed in a mouse serum transfer model of inflammatory arthritis [53]. Importantly, the inflammatory responses are reduced in mice with 5-LOX and leukotriene A4 hydrolase enzyme deficiency [54]. In addition, another lipoxygenase type, namely 15-lipoxygenase, is also involved in the pathogenesis of rheumatoid arthritis via the NF-κB pathway. It has been described that the 15-lipoxygenase metabolite, 15-(*S*)-HETE increases the IκBα degradation and the nuclear translocation of NF-κB subunit [48]. It has been observed that the NF-κB pathway is activated in the early stage of joint inflammation and NF-κB DNA binding activity is increased in rheumatoid arthritis patients [55]. These results indicate NF-кB activity and LOX activity are also closely linked in rheumatoid arthritis and that inhibition of lipoxygenases could also find a therapeutic application in this field. 

### 5.4. Inflammatory Bowel Disease

The role of leukotrienes in inflammatory bowel disease (IBD) has been explored. A colonic biopsy test from patients with IBD showed 3-7 fold enhancement of 5-lipoxygenase, FLAP and LTA4 hydrolase expression in the colonic mucosa and the rectal dialysates, which form the cellular basis for LTB4 synthesis [56]. More recently, Cys-leukotiene E4 (LTE4) was considered as a biomarker for IBD since the urinary excretion of LTE4 was significantly increased in patients with IBD [57]. All these data together suggest that inhibition of lipoxygenase activity and leukotriene bio-synthesis can be a valuable approach for treatment of such inflammatory diseases.

### 5.5. Lipoxygenase in Cancer

Lipoxygenases and their catalysis products are associated with carcinogenic processes such as tumor cell proliferation, differentiation, and apoptosis [58]. Several lines of evidence have proven the crucial role of lipoxygenases in cancer. In human prostate cancer cells, the overexpression of platelet 12-lipoxygenase (p12-LOX) has been observed, which is a trigger for angiogenesis and tumor growth [59]. The increased expression of the 5-LOX enzyme and the LTB4 receptors were observed in pancreatic cancer. In addition, 5-LOX expression levels were suggested as indicator for early neoplastic lesions [60]. Leukotriene LTB4 is a potential stimulator for cancer cell growth and also plays a role in the formation of ROS in response to hypoxia [60,61]. It has also been shown that the 5-LOX metabolite LTB4 is capable of activating the transcription factor NF-κB in cancer cells, which suggest a tumor promoting role via this route [49]. The roles of 15-LOX-1 metabolites are reported in the development of breast cancer by promoting the invasion of tumor cells into the lymphatic vessels and the formation of lymph node metastasis [62]. In colon cancer cells it has been shown that 15-LOX-1 expression suppresses the metastatic phenotype of these cells [63] and this enzyme is linked to increased NF-κB transcriptional activity [46]. Contrary to a tumor promoter role of 15-LOX-1 a tumor suppressor role of 15-LOX-2 has been described in prostate cancer [18,64]. For 15-LOX-2, however, no connection with NF-κB signaling has been described so far. These studies indicate that the lipoxygenase expression is associated with the development of cancer. For 5-LOX and 15-LOX-1 the activity is linked to NF-κB activity, whereas such a connection has not been described for the other lipoxygenases. Taking all this evidence together, lipoxygenases are an emerging group of cancer targets. 

## 6. Biosynthesis of Lipoxins: Termination of Inflammatory Responses

**Scheme 2:**
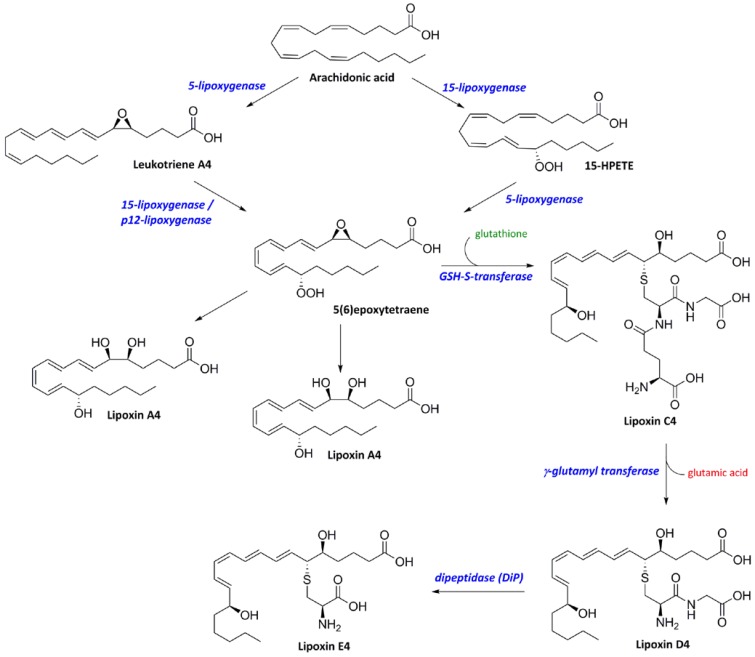
Two lipoxygenase-based synthesis routes of lipoxins (LXs).

Within the eicosanoid cascade, lipoxins that are formed by lipoxygenases have potential as counter-regulator to resolve inflammation and to restore cellular homeostasis. Lipoxins (LXs) are generated from arachidonic acid through two lipoxygenase-based synthesis routes. The first route involves the formation of LTA4 by 5-LOX and the conversion of LTA4 to the intermediate 5(6)-epoxytetraene, which is subsequently converted into LXA4 and LXB4. The second route for LXs formation is initiated by 15-LOX activity to oxidize arachidonic acid to 15-HPETE then followed by 5-LOX activity, which convert 15-HPETE to 5(6)-epoxytetraene [65]. Both routes, which are involving 5-LOX activity in the lipoxin production, show that 5-LOX activity is important, not only in the formation of pro-inflammatory mediators, but also in the formation of anti-inflammatory mediators. Moreover, like the leukotrienes, an addition of glutathione (GSH) by GSH-S-transferase activity generates cysteinyl lipoxin LXC4. LXD4 and LXE4 are generated in a similar manner as in the leukotriene biosynthesis pathways (Scheme 2). 

Only a few explorations on LXC4, LXD4, and LXE4 have been done and their biological roles have not been investigated in detail. However, it has been reported that LXC4, LXD4, and LXE4 are selectively generated by eosinophils and not by neutrophils and platelets [65]. LXA4 and LXB4, with LXA4 being the most studied, are emerging as mediators to stop the inflammatory responses and to switch the cells to normal homeostasis [66]. LXA4 and LXB4 actions in cells and tissues are mediated through their interactions with lipoxin receptors. The lipoxin A receptor (ALXR) transmits stop signals to reduce the pro-inflammatory signals to terminate neutrophil migration. Furthermore, it stimulates the activation of monocytes and macrophages, and inhibits the leukotriene B4 formation [67]. In addition, LXA4 can also act as a partial agonist for the LTD4 receptor by blocking the LTD4 binding [65]. LXA4 stimulated-ALXR is able to block the NF-κB-mediated gene expression and inhibits the degradation of IκBα [66,68].

Lipoxins production, which is related to the activity of 5-, p12-, and 15-LOXs, has been proven to be important and the alteration of the enzyme activity determines the levels of lipoxin [69,70]. Up-regulation of arachidonate 15-lipoxygenase genes has been reported in the gene profiling of glucocorticoid-treated nasal polyps [71], which is also an indication of 15-HPETE production during the termination of inflammatory process. Another study on the blood polymorphonuclear cells (PMN) from asthmatic patients shows an increase of lipoxin production together with the activation of 5-lipoxygenase [72]. In addition, aspirin, a non-steroidal anti-inflammatory drug which inhibits the activity of pro-inflammatory eicosanoids produced by cyclooxygenase (COX), triggers the biosynthesis of LXA4 and the 15-epimer of LXA4 accompanied by the increase of brain 5-LOX activity in rat infused with lipopolysaccharide (LPS) [73]. Taking all these findings together, these studies indicate that the increase of 5-LOX activity does not solely contribute to the production of leukotrienes but also to the increase of lipoxin levels. Since the 5-LOX activity is important for both initiation and termination of inflammation, the modulation of this enzyme is crucial for inflammation therapy. Furthermore, its dual functions in the inflammatory processes make 5-LOX an interesting enzyme for further investigation of both inhibitors and activators.

## 7. Lipoxygenase Inhibitors

**Figure 2:**
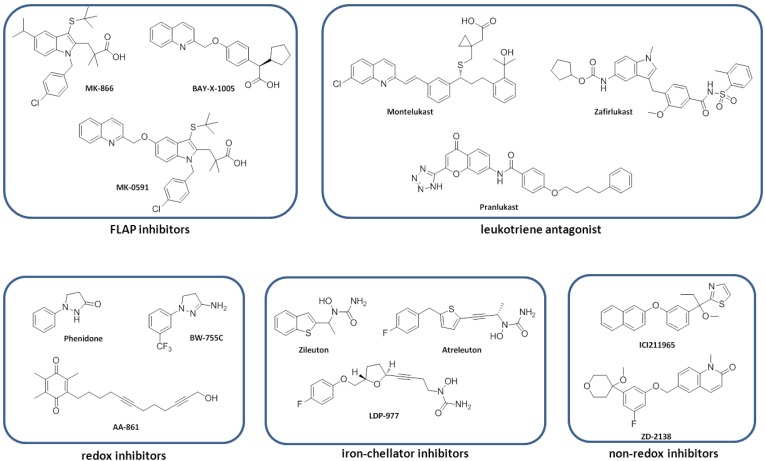
Lipoxygenase inhibitors.

Considering the potent pro-inflammatory properties of lipoxygenases and their products, the modulation of the lipoxygenase pathways using small molecule inhibitors should provide new therapeutic approaches for numerous inflammatory diseases and cancer. Various approaches have been developed to inhibit lipoxygenases. Several synthetic small molecules as well as isolated natural compounds have been tested for the inhibition of lipoxygenases (Figure 2). Recently, it was reported that Δ^9^-tetrahydrocannabinol (Δ^9^-THC), which is an active component extracted from cannabis, shows an inhibition of 15-lipoxygenase with an IC_50_ of 2.42 µM [74]. Nordihydroguaiaretic acid (NDGA), which is a well-known antioxidant, inhibits platelet 12-lipoxygenase and 15-lipoxygenase [75]. Another compound with iron binding properties; 4-(2-oxopentadeca-4-yne)phenyl propanoic acid (OPP) (Figure 2), shows a mixed type inhibition towards leukocyte 12-lipoxygenase with K_i_ and K_i_' values respectively are 0.2 µM and 4.5 µM [76]. The natural product curcumin, which is found in turmeric, is a modulator of arachidonic acid metabolism through the 5-LOX pathway [77].

The role of 5-lipoxygenase in inflammation has been intensively investigated. The fact that the mechanism of 5-LOX activation is more complex compared to the others lipoxygenases, opens opportunities for alternative strategies of inhibition. Inhibitors for leukotriene biosynthesis via 5-lipoxygenase can be divided into FLAP inhibitors, redox inhibitors, non-redox inhibitors, and iron-chelator inhibitors (Figure 2).

### 7.1. FLAP Inhibitors

Compound MK-866, which was introduced by Gillard *et al.*, is a potent leukotriene biosynthesis inhibitor [78]. This compound selectively inhibits FLAP without affecting 5-LOX, or phospholipase in the leukotriene biosynthesis pathway [78,79]. MK-866 was shown to be safe for consumption and has an effect on the early and late stages of asthmatic responses to allergens [80]. The other compounds that belong to the FLAP inhibitor class are MK-0591 and Bay-X-1005 (Figure 2). These compounds show a potent leukotriene inhibition in the nanomolar range [81,82]. However, the presence of arachidonic acid and other *cis*-unsaturated fatty acids in blood can compete with those inhibitors for FLAP binding, causing a low inhibitors efficacy in whole blood assay [83]. This results in a 50–200 fold reduction in potency in whole blood assays in comparison with assays in isolated leukocytes [81,82]. This reduced efficacy for FLAP inhibition in excess of arachidonic acid indicates that inhibition of FLAP in the leukotriene biosynthesis pathway might be less effective [84].

### 7.2. Redox Inhibitors

Redox inhibitors basically act as antioxidants for the oxidation reaction performed by lipoxygenases. The redox inhibitors phenidone, BW755C, and AA-861 are well known as reducing agents (Figure 2) [85,86]. Structure activity relationships for this class of inhibitors are relatively difficult to describe. Nevertheless, it has been recognized that, apart from the redox potency [87], lipophilicity is also important [86]. Recently, a new redox inhibitor for 5-LOX has been reported, which is a trimer or tetramer of caffeoyl clusters (Figure 2), with IC_50_ values of 0.79 µM and 0.66 µM, respectively [88]. Furthermore, redox inhibitors have a low selectivity for 5-LOX inhibition compared to COXs inhibition [85]. Although they display a high potency to inhibit leukotriene biosynthesis, an interference with other biological redox processes has been reported. The formation of methaemoglobin is one of the problems that were reported upon application of redox inhibitors [89]. 

### 7.3. Iron-Chelator Inhibitors

In general a non-heme iron atom in lipoxygenases coordinates with amino acid residues and a water molecule forming an octahedral complex [90]. The coordinated water molecule in the active site is stabilized by a hydrogen bond with the carboxylate of an Ile residue. The iron atom in the 12-lipoxygenase active site is more ordered in comparison to 5- or 15-lipoxygenase. The water molecule in 5-lipoxygenase still coordinates with the iron atom but is slightly off the position to form an octahedral complex, while in contrast no water molecule is coordinated with the iron atom in the 15-lipoxygenase active site. Besides coordinating with a water molecule, in 5-lipoxygenase the iron atom coordinates with three His residues, and one Asn, whereas in 12- and 15-lipoxygenases four His residues with one Ile are coordinated with the iron [91]. The crystal structure of the enzymes with their iron complex provides an understanding about the regio- and stereoselectivity of the catalytic reaction, which is important for the development of inhibitors of the iron-chelator class. 

Inhibition of 5-LOX can be achieved by replacing one of the iron ligands with a small molecule ligand to create a complex. Molecules with iron-chelating functionalities such as hydroxamic acid or N-hydroxyurea are potent inhibitors for 5-LOX (Figure 2) [92]. Zileuton is one of the 5-LOX iron-chelator inhibitors that is already on the market for the treatment of asthma. In a number of clinical trials, zileuton has been shown to improve airway function and reduce the asthmatic symptoms as well as the inflammation in the respiratory system. Despite its effectiveness, zileuton is not the first choice therapy due to its side effect such as nausea and idiosyncratic effects on the liver [93]. Further development of this class of inhibitors led to the identification of atreleuton, which inhibits LTB4 and cys-LTE4 production and has a potency that is about 5-fold enhanced in comparison to zileuton [94]. Atreleuton, which has entered clinical trials for atherosclerosis and cardiovascular diseases, is one of the leading 5-LO inhibitors in clinical development [95]. Another N-hydroxyurea derivative, CMI-977 (LDP-977) [96] showed potency as a new drug for asthma by suppressing 5-LOX activity in blood and also by inhibition of anti-IgE-induced contractions of the airway tissue [97,98]. These studies suggest that the development of iron-chelator inhibitors for lipoxygenases could be an interesting concept for further exploration. 

### 7.4. Non-Redox Inhibitors

Non-redox inhibitors do not interfere with the oxidation reaction of lipoxygenases or have apparent iron-binding properties. Inhibition of the enzyme activity can take effect by competitive binding to the active site or by binding to an allosteric binding site that regulates the activity of the enzyme. The (methoxyalkyl)thiazole (ICI211965) (Figure 2) selectively inhibits 5-LOX activity, which reduces LTC4 and LTB4 synthesis in animal and human blood samples [99]. Unfortunately, steady-state kinetic analyses of this compound for 5-LOX have not been successfully performed and therefore it has not been possible to determine whether the inhibition is competitive with the substrate arachidonic acid or not [100]. Although, ICI211965 is a highly potent 5-LOX inhibitor from a novel structural class, it has been reported to have a low oral potency. The methoxytetrahydropyran compound ZD-2138 (Figure 2) shows an improvement of the oral potency compared to ICI211965 for the treatments of arthritis and asthma [101]. Furthermore, ZD-2138 inhibits antigen-induced leukotriene release at the micromolar concentration range [102]. However, the results from a clinical trial for its application as an anti-arthritis agent were disappointing and therefore research on this molecule was discontinued [103]. Interestingly, recently a compound class containing a salicylate core structure has been identified to inhibit or activate lipoxygenases presumably via an allosteric mechanism [104,105,106]. 

### 7.5. Leukotriene Antagonist

Recently, leukotriene receptor antagonists have appeared as a class of compounds that have superior properties for suppression of leukotriene biosynthesis. Pranlukast, zafirlukast and montelukast (Figure 2), three of the leukotriene receptor antagonists, have also shown good efficacy in the treatment of asthma [107,108]. These drugs block the binding of leukotriene D4 and also LTC4 and LTE4 to the CysLTR1 in the lungs and bronchial tubes, which resulted in the reduction of airway constriction, and mucus accumulation in the lungs and airways. Interestingly, it has also been reported that montelukast possess secondary anti-inflammatory properties to inhibit the activity of 5-LOX and HATs [109]. Montelukast suppresses the leukotriene biosynthesis by selective inhibition of 5-LOX and gives no effect on the other enzymes involved in the leukotrienes biosynthesis pathway such as LTA4 hydrolase and LTC4 synthase [110]. Moreover, montelukast alters the activity of the NF-κB transcription factor p65-associated HAT activity and reduces the TNF-α-stimulated IL-8 expression [111]. However, it has been reported that the usage of this leukotriene antagonist produces neuropsychiatric side effects which is a major concern for its safety. 

## 8. Conclusions

Lipoxygenases are an intensively studies class of enzymes and an increasing number of functions in various diseases are being found for their lipid metabolites. Although lipoxygenases has been recognized classically as drug targets for treatment of inflammation more recently anti-inflammatory effects have been discovered for the lipoxins, which are also lipoxygenase metabolites. Interestingly, also connections between lipoxygenases and diseases such as cancer and atherosclerosis have been identified. There is a limited amount of data on the connections between lipoxygenase metabolites and signal transduction pathways such as the NF-κB pathway. Taken this all together, literature demonstrates a key regulatory role for lipoxygenases and their metabolites in many physiological processes, which positions them at the center of many disease models. Nevertheless, their versatile roles and their connection to signaling cascades indicates that it can be difficult to redirect specific physiological processes using small molecule inhibitors of lipoxygenases. 

A variety of compounds have been introduced to modulate lipoxygenase enzyme activity and ultimately to provide new drugs for inflammation. Despite of their high potency to inhibit leukotriene production, their limitation in efficacy in specific disease models are still a concern that needs to be resolved. In view of that the development of lipoxygenase modulator with an improve potency and selectivity for specific therapeutic applications as well as novel methods to study the functional consequences of these oxidative enzymes remains an important challenge.
